# “We Have a Lot of Power…”: A Medical School’s Journey Through Its New Deferred Action for Childhood Arrivals (DACA) Initiative

**DOI:** 10.7759/cureus.6037

**Published:** 2019-10-30

**Authors:** Katherine Wasson, Cynthia Chaidez, Lena Hatchett, Belsy Garcia Manrique, Emelin Garcia Nieto, Aldo Martinez, Mark G Kuczewski

**Affiliations:** 1 Bioethics and Medical Education, Loyola University Chicago, Stritch School of Medicine, Maywood, USA; 2 Medical Education, Loyola University Chicago, Stritch School of Medicine, Maywood, USA; 3 Bioethics and Medical Education, Loyola University Chicago, Stritch School of Medicine, Maywood, USA; 4 Family Medicine, University of Illinois, Chicago, USA; 5 Pediatrics, Emory University, Atlanta, USA; 6 Family Medicine, Loyola University Chicago, Stritch School of Medicine, Maywood, USA

**Keywords:** undergraduate medical education, medical students, deferred action for childhood arrivals (daca), policy change, resilience

## Abstract

Purpose

To describe and analyze qualitatively the impact of implementing the “Stritch Deferred Action for Childhood Arrivals (DACA) Initiative” (SDI) at the Stritch School of Medicine (SSOM), Loyola University, Chicago in 2012. The SDI is a three-step process that included: 1) opening the Stritch admissions policy to welcome DACA students to apply, 2) evaluating DACA applicants equitably with all other applicants, and 3) seeking funding to enable these students to matriculate.

Method

Focus groups and in-depth interviews were conducted to explore DACA and non-DACA students’ experience of the SDI on their medical school journey and the institutional culture. During the study (in 2017-18), the medical school year (M)1-M3 cohorts included DACA students, while the M4 class did not. A grounded theory method was used to summarize and analyze qualitative data.

Results

Four major themes and 11 subthemes emerged from the data analysis. "Beliefs and Attitudes" included the subthemes of motivation to become physicians, resilience, and the mission and values of individuals and the institution. Students noted "obstacles" in reaching medical school, along with those they encountered within it. They also noted multiple "opportunities" presented through the SDI and the importance of mentors and allies. Lastly, the "impact" of the SDI on individuals, the institution, and the wider community was discussed by participants.

Conclusion

Enacting the SDI enabled cohorts of DACA recipients to matriculate at SSOM. Both DACA and non-DACA students in this study identified the importance of including these students as future physicians and articulated the impact of this change on them, their classmates, the institution, and the community as solidarity was formed and students' awareness of their power as future physicians to advocate for underserved populations developed.

## Introduction

In 2012, former President Barack Obama enacted the Deferred Action for Childhood Arrivals (DACA) program. It permitted persons brought into the United States (US) prior to the age of 16, who typically either lacked proper immigration documentation or overstayed a visa, to apply for deferral of action on their immigration status and receive a work permit. This deferral enabled them to work and improved the prospects for attending medical school since they could now seek medical licensure in most states [1-2]. Once this national policy change was made, the Stritch School of Medicine (SSOM) of Loyola University Chicago enacted what we call the “Stritch DACA Initiative” or SDI, a three-step process to 1) open the Stritch admissions policy to welcome DACA students to apply, 2) evaluate DACA applicants equitably with all others, and 3) seek funding to enable these students to matriculate, as they are not eligible for federal loans. As a Jesuit institution, our mission to promote social justice and serve the underserved guided the policy initiative and actions [2]. At the time of this study, Stritch had three cohorts of DACA recipients, ranging from seven to 14 students per class, uniquely positioning us to study the impact of this initiative on the experience of all medical students and the institutional culture.

In general, there was a climate of support for this initiative which helped to gather partners to provide financial aid. The initial cohort of seven students received loans from the state’s infrastructure bank [3]. This loan program was facilitated by a member of the University’s Board of Trustees who also sat on the board of the infrastructure bank. Similarly, Trinity Health, which owns the Loyola University Health System, provided some loans for additional cohorts. The medical school and the university must continually locate funding either internally or extramurally for each new cohort, again demonstrating the ongoing commitment of senior leadership over a period of years.

## Materials and methods

Six focus groups and seven in-depth interviews were conducted to explore DACA and non-DACA students’ experience of the SDI on their medical school journey and the institutional culture at the medical school. Conducted in 2017-18, the medical school year (M)1-M3 cohorts included DACA students, while the M4 class did not. A grounded theory method was used to summarize and analyze qualitative data. This approach is frequently used in qualitative studies as “hypothesis-seeking,” particularly where little is known about a phenomenon [4-5]. The basic premise of this approach is to generate theory from data rather than impose a preexisting theory or model [6]. Students were asked to describe their experience of the DACA initiative and how it affected the SSOM environment and their experience of medical education.

Participants

Eighteen DACA students and 16 non-DACA students from all four years of the medical school participated in focus groups and in-depth interviews. The participants were female (50%) and identified as Hispanic (41%), White (26%), Asian (24%), and Black/African American (6%). Most identified their religion as Catholic, Protestant, or Christian (62%) with 24% selecting “None.” The study was approved by the Institutional Review Board at Loyola University Chicago and all students consented.

Analysis

Focus group discussions and in-depth interviews were conducted by a trained facilitator, tape-recorded, and transcribed. The first three focus group transcripts were read by all authors to generate labels by sorting and summarizing concepts into codes using a grounded theory approach [6]. The study team wrote memos, ideas, and notes about each code comparing how codes related to one another to represent a repeated pattern and story. The iterative process allowed the team to discover patterns and pathways consistent with the emergent strategies approach in organizations [7]. Major themes were summarized to establish clear links between the research questions and the raw data [8]. Once the codebook was developed, three members of the research team (KW, CC, LH) coded all the transcripts. When there was a disagreement over patterns and themes, all three reviewers discussed the theme until there was a consensus. The process yielded four major themes, 11 subthemes, and 29 codes.

## Results

The data analysis produced four major themes: beliefs and attitudes, obstacles, opportunities, and the impact of the DACA Initiative. These major themes interact with one another, the sub-themes, and codes in each section in a non-linear way. They inform and impact one another throughout this journey to and through medical school for these students.

Beliefs and attitudes

Beliefs and attitudes express the students’ values and shape their interactions and how they respond to challenges. The students’ responses captured the sub-themes of motivation, resilience, and mission and values in their medical school journey leading up to their experience of SSOM and beyond in some cases. The interaction of these themes and sub-themes is not linear, as beliefs and attitudes interact with motivations and mission and values, and vice versa. Furthermore, the lens of resilience was used to frame parts of the journey and was also informed by the beliefs and attitudes of the students.

Motivation

Both DACA and non-DACA students expressed a clear motivation to become physicians and a strong commitment to this vocation. There were no noticeable differences in the early motivation between these students, which included a recognition of their vocation and awareness of their role as physicians. Respondents describe personal interests and the needs of the family as key motivations in pursuing their vocation often defined by a key event, e.g., sick relative or receipt of quality care.

DACA: “….I want to be in science. I want to be in healthcare and I, in one way or another, want to work with underserved populations, which at that time for me was my family that was in another country without the resources that they needed.”

Non-DACA: “I decided to become a doctor because I wanted to help people and I felt like being a physician was a very tangible way to make a difference in the world in a more, like, immediate fashion…”

Resilience

Resilience, the learned skill of coping and adapting to adversity, was vital as the students faced multiple obstacles in pursuing their calling to become physicians. Students’ responses highlighted the importance of adaptiveness, vulnerability/empowerment, gratitude, expectations, and hope/optimism in their medical school journey.

DACA: “Aside from the never giving up, constant resiliency, just finding that inner motivation … so I would say find your own -- like, find a path and make it happen. Find the resources that you need, reach out to somebody.”

DACA: “…this is not to say it in a harsh way, but no one promised us that life will be easy and you just have to take it. You have to accept whatever is coming your way right now and just stay positive throughout the whole thing. Your faith and your determination is one of the most important things that will get you through it.”

Mission and Values

Mission and values are key beliefs and attitudes, as they are fundamental drivers in what is important to a person and an institution. The mission is at the core of an institution: its purpose. Individuals may also have a mission in life: a “raison d’etre” which informs their choices and actions [1]. Loyola University Chicago’s mission statement (http://www.luc.edu/mission/index.shtml) states, “We are Chicago’s Jesuit, Catholic University: a diverse community seeking God in all things and working to expand knowledge in the service of humanity through learning, justice, and faith.” The sub-theme of mission and values applies to the broader university and medical school, as it opened its doors to DACA applicants based on its Jesuit mission and values, and the students, faculty, and staff who facilitated and engaged with that change. Student responses highlighted the contributions of spirituality, justice/injustice, and leadership to their experience in medical school.

Non-DACA: “…I've always thought of it as kind of the Jesuits really urge you to become educated in line with that (being) men and women for others, so you use kind of like the privilege of your education to help others and I feel like as physicians that's really something that we're called to do….”

Obstacles

The second major theme was obstacles on this journey, which included barriers, the emotional impact and assumptions, and biases about race and diversity. For many DACA students, the emotional barriers included the uncertainty of their prior undocumented status, the accompanying fear and anxiety, and its impact on their mental health and others. Practical barriers included not being able to obtain a driver’s license or be eligible for financial aid. 

DACA: “I think if I had to go back and change my past I don't think I would. I think I would actually be okay with having to go back to the same path I had to take because I think it's really shaped who I am. It's shaped my ideals. It's shaped the kind of physician and the kind of person I want to be and…although there were many times where it was extremely frustrating, a lot of tears, a lot of anger, I really don't regret any of it and I don't have anyone to blame for this. I think it's actually been very humbling and beautiful…I think that that's taught me…a lot about what I can do and the kind of abilities that I have as a human being.”

Non-DACA students shared their growing awareness of this journey and its challenges for their DACA classmates:

Non-DACA: “You just like appreciate the fact that people -- like, we're all on the same (path)… doing the exact same stuff pretty much, while people have … a hard slog, even harder than it already is just to get here.”

Students recognized the existence of assumptions, and sometimes misperceptions, about race and diversity and reflected on their impact on individuals and the institutional community.

Non-DACA: “…I think that the value of the DACA students being integrated into the school comes in…it comes in maybe changing the views and mindsets of some of these people that we've been talking about, like, the attending who is skeptical about the capacity of the academic credentials or the resident who has some kind of less informed views…”

Opportunities

Student responses described the diverse benefits of the SDI both now and for the future. Students talked about opportunities due to the SDI in broad terms, including the benefits of the DACA students, allies, and the role of resources and education in this journey. Non-DACA students described how having DACA students as classmates gave them first-hand knowledge of the challenges the latter and their communities faced. Non-DACA students became more aware of the stories and realities of their DACA classmates. In spite of having varied pathways to medical school, once here, they had many shared experiences. Non-DACA students articulated their appreciation of the opportunity to learn from their DACA classmates in the present and apply those insights in the future.

Non-DACA: “… I think ideally our population of physicians should represent our population of patients which is one of the great things about DACA students and diversity and I think they are better -- will always be better equipped to take care of people who are in their community than I am, but at least I hope to learn from them and be able to kind of translate that into caring for different communities…”

Impact of the policy change at SSOM

The impact of the SDI was felt and experienced by students on multiple levels, including the individuals, community, and institution. The students were aware of and reflected on the benefits of the SDI on these levels and for themselves.

Impact on Individuals and Community

Students reflected on the impact of the SDI and training with DACA recipients to become physicians and described the significance of being first-generation medical students and the accompanying power and responsibility. They also noted the opportunity for SSOM to be an example to other schools and through this process, the SDI represented the outward-facing Stritch Jesuit identity.

DACA: “…I think just like Stritch has changed my life. I want to say that us, DACA individuals, have changed the Stritch community and even the Loyola community. I think the most gratifying part of this whole process really has been the community component which is, you know, as physicians, I think we have a lot of power in empowering and advocating for underserved populations….”

DACA: “We have a lot of power with what we're going through right now because we are one of the first generations to kind of go through this hurdle and just being an example and a role model, not just to other classmates but to other institutions, I think says a lot.”

Impact on the Institution

The SDI helped to transform the culture of SSOM from having a commitment to values to acting on those values. The culture at SSOM was affected by the SDI since having DACA students integrated into the school became “normal” and their experience more well-understood by classmates and solidarity with them was formed over time. Both groups experienced raised awareness of this solidarity and began to recognize their power as future physicians and their correlative responsibility to advocate for others. Students described this process of change through the importance of communication, advocacy, curriculum, and political climate. The change happened over time and was, therefore, uneven, with different groups and parts of the institution learning about the SDI rationale at different points and understanding it or not.

Communication from the medical school was not always perceived to be consistent among the different cohorts of students and the wider Loyola community. The administration provided information sessions to address the policy changes, which were seen as partly successful, although students thought it was harder to convey this information in the hospital setting than in the medical school, given the number of sites, faculty, and staff involved. The medical school had less control over the messaging outside of its walls. As a consequence, there may have been misperceptions about the SDI and DACA students in some areas of the institution.

Non-DACA: “I think that the institution didn't do a good enough job of making sure everyone understood what was happening and, like -- because the people who are going to end up going to those sessions that really explain the policy are going to overwhelmingly be people who are probably basically like oh yeah, I'm for that.”

Non-DACA: “…I think like with anything there are pockets of people, and not just in the medical school, but like faculty, and you just hear from different pockets that all this kind of rumors that like it is free for them (DACA students) and taxpayer money is going to this or that, they could have really, really low Medical College Admission Test (MCAT) scores and still get into Loyola, they're getting like this preferential treatment above everyone else…and I think all those rumors kind of flying around there were certainly like mixed feelings. But I think largely it felt celebratory was my feeling of it.”

A few students felt that the SDI raised divisions within the SSOM culture because of the national political views for or against the immigration policy and SSOM culture of open discussion about the meaning and values of culture, diversity, and justice. This was an interesting reflection because students stated that the SDI was an opportunity to use their power and privilege as a medical student and future physician to act in ways to have a “bigger voice” greater than oneself.

Non-DACA: “I've thought about this more recently, as a med student, is that the position and your status as a physician give you this type of power and ability and it's…your public voice, and it's kind of your responsibility…”

Non-DACA: “…I'm really glad our school made the move that it did…our patients need DACA students and need DACA physicians and I just hope that no matter what legislation happens…that our school community will continue to…do what it needs to do no matter how political that might be to make sure that DACA students are continued to, like, become physicians.”

Benefits of the SDI

Benefits of the SDI were felt and illustrated on a personal level, sometimes expressed as learning opportunities by the students and for the institution. Students captured how the SDI was received and largely embraced and the climate of inclusion and solidarity it created for all students which built on institutional values.

DACA: “…last year I was really touched because one of the faculty and the staff said, well, we should be the ones thanking you (DACA students) because you have changed the face of the institution and a lot of how our classmates even interact with each other. And I think that is above and beyond any expectation that you would ever have, at least from my background of being really quiet about my immigration status and shameful, but not in a purposeful way, just because of fear and anxiety. So that expectation has been above and beyond and it's been quite nice to see that.”

DACA: “…I knew there was barely…any student that was undocumented that was a medical student, so having the fact that there were seven of us was definitely something very enriching, and also just being surrounded by faculty and attending doctors who really understood the purpose of having Stritch support DACA students was something that I wasn't really prepared for, something that left a really lasting impression on me.”

Non-DACA students recognized that having DACA students as classmates was a benefit to them, specifically a growth opportunity for understanding diversity and justice. Students spoke in general terms about solidarity with and for DACA students and immigrant rights in the current political climate. The SDI created an opportunity to maintain and foster a sense of collective awareness and a commitment to support justice now for the underserved and immigrant communities in the future. Others described the SDI as a “natural experiment” on diversity that allowed peers to question assumptions about diversity beyond race and the meaning of professional obligations to mobilize for justice in the medical school.

Non-DACA: “The majority of the school had no idea they were DACA students because they're just so integrated into who we are.” 

Non-DACA: “I think you get the sense that all of us feel a sense of responsibility for our classmates…feel like whenever their sense of safety is threatened that our sense of safety in a sense is threatened and that we have a right and a responsibility to do something.”

## Discussion

This qualitative study explored the impact of the SDI on DACA and non-DACA students at SSOM. Students described their experience of medical school after this change and its impact on them, the culture at SSOM, and beyond. 

As we coded and analyzed these data, a clear narrative of a journey emerged from the participants, both DACA and non-DACA. As a team, we saw that the relationship between the concepts and codes evoked the image of a river. The metaphor of a river describes the journey of these students well because it is not linear and can be reshaped, which is what the admission of DACA recipients did to the individuals and culture at SSOM. The current of a river is a natural force that propels the water forward. It is present at all times, to a greater or lesser degree, with ebbs and flows, and ultimately helps determine the path of the river over time. In this narrative, the current of the river is the SDI and includes the theme of beliefs and attitudes which shaped the journey and experience of the students. Rivers include obstacles and barriers, e.g. rocks, which may hinder the journey. The river ebbs when faced with stones and obstacles and the current is less forceful or clear in its direction. The flow of the river is when the current is moving forward at a slow or quick pace. While it can flow at different rates, this theme captures the opportunities the students had during their journey to and through medical school. Finally, a riverbed can be reshaped. Here, the implementation of the SDI facilitated the journey of the DACA students and reshaped the pathway for the DACA students, their non-DACA peers and faculty, and those who follow. 

Individual-level: resilience

For DACA recipients in this study, national policy change permitted them to obtain DACA, and the SDI opened a pathway that had been closed to them. They could pursue their calling to become physicians. Resilience was vital to their journey and ability to respond to this new opportunity and others during medical school. The non-DACA students reflected on the increased awareness of their responsibility and power as future physicians to advocate and stand in solidarity with DACA students and underserved communities for social justice. The lives and pathways of both groups of students were changed because of this exposure to DACA recipients, their stories, aspirations, and achievements.

Interpersonal level: courage of the institution

In keeping with its Jesuit values, SSOM took the courageous step to enact the SDI, open its admissions process to DACA recipients, publicize that change, help obtain funding, and welcome these students. SSOM did not limit the number but allowed these students to compete equally with other applicants. The result was cohorts of seven to 14 DACA recipients during this study. We hypothesize that having a cohort of DACA students and being public about their inclusion had a larger impact on the culture of the institution than admitting one to two DACA students per year would have [9]. Sessions were held to inform the student body about the SDI and rationale. While these were not uniformly attended or accepted, they likely did raise awareness of the changes and attempted to address assumptions and meanings about race, diversity, and inclusion of DACA students. The cohort approach also drew attention from the media and contributed to SSOM becoming a national advocate for DACA medical students. It allowed SSOM to be a model for other educational institutions.

Community-level: richness of communities

Multiple students noted the importance of training physicians who reflect the diverse communities in the United States. Some, though not all, DACA students described coming from families with limited resources and wanting to serve those communities in the future. A related study described the barriers, along with the education to career pipeline for underrepresented minorities [10]. Of note, DACA students in our study described parents, teachers, and others as mentors who helped create opportunities to pursue the medical school journey despite the difficulties and uncertainty. Families and communities had enabled them to reach medical school and they wanted to complete the circle by giving back as physicians. Non-DACA students recognized the importance of training physicians with a heart for the diverse and underserved communities; they were inspired by their DACA classmates’ commitments to these groups and believed their solidarity with their classmates and a better understanding of the challenges they faced would help them relate to similar patients in future.

Limitations

This was a cross-sectional study capturing those students at SSOM in 2017-18. As in all studies, those who participate are self-selecting and may have particular views they want to share. Here, those who may have strong views against the policy change may not have felt free to share those views in this context. Qualitative studies are not meant to be generalized, but rather to generate theory. Our results need to be investigated further.

## Conclusions

In conclusion, enacting the SDI enabled cohorts of DACA recipients to matriculate at SSOM. This change created opportunities to build on our Jesuit mission, identity, and values. Students reflected on the influence and impact of this change, which was bi-directional as the stories and journeys of the DACA students influenced other students, faculty, and staff and vice versa. Students noted the SDI and its impact enriched their medical school journey and training as future physicians as they learned from one another. 
